# Audio-/Videorecording Clinic Visits for Patient’s Personal Use in the United States: Cross-Sectional Survey

**DOI:** 10.2196/11308

**Published:** 2018-09-12

**Authors:** Paul J Barr, Kyra Bonasia, Kanak Verma, Michelle D Dannenberg, Cameron Yi, Ethan Andrews, Marisha Palm, Kerri L Cavanaugh, Meredith Masel, Marie-Anne Durand

**Affiliations:** 1 The Dartmouth Institute for Health Policy & Clinical Practice Geisel School of Medicine at Dartmouth Dartmouth College Lebanon, NH United States; 2 Geisel School of Medicine at Dartmouth Dartmouth College Hanover, NH United States; 3 University of St. Andrews Fife United Kingdom; 4 Vanderbilt Center for Effective Health Communication Vanderbilt University Medical Center Nashville, TN United States; 5 Division of Nephrology and Hypertension Department of Medicine Vanderbilt University Medical Center Nashville, TN United States; 6 Oliver Center for Patient Safety & Quality Healthcare University of Texas Medical Branch Galveston, TX United States

**Keywords:** audiorecording, health care, health system, policy, United States, videorecording

## Abstract

**Background:**

Few clinics in the United States routinely offer patients audio or video recordings of their clinic visits. While interest in this practice has increased, to date, there are no data on the prevalence of recording clinic visits in the United States.

**Objective:**

Our objectives were to (1) determine the prevalence of audiorecording clinic visits for patients’ personal use in the United States, (2) assess the attitudes of clinicians and public toward recording, and (3) identify whether policies exist to guide recording practices in 49 of the largest health systems in the United States.

**Methods:**

We administered 2 parallel cross-sectional surveys in July 2017 to the internet panels of US-based clinicians (SERMO Panel) and the US public (Qualtrics Panel). To ensure a diverse range of perspectives, we set quotas to capture clinicians from 8 specialties. Quotas were also applied to the public survey based on US census data (gender, race, ethnicity, and language other than English spoken at home) to approximate the US adult population. We contacted 49 of the largest health systems (by clinician number) in the United States by email and telephone to determine the existence, or absence, of policies to guide audiorecordings of clinic visits for patients’ personal use. Multiple logistic regression models were used to determine factors associated with recording.

**Results:**

In total, 456 clinicians and 524 public respondents completed the surveys. More than one-quarter of clinicians (129/456, 28.3%) reported that they had recorded a clinic visit for patients’ personal use, while 18.7% (98/524) of the public reported doing so, including 2.7% (14/524) who recorded visits without the clinician’s permission. Amongst clinicians who had not recorded a clinic visit, 49.5% (162/327) would be willing to do so in the future, while 66.0% (346/524) of the public would be willing to record in the future. Clinician specialty was associated with prior recording: specifically oncology (odds ratio [OR] 5.1, 95% CI 1.9-14.9; *P*=.002) and physical rehabilitation (OR 3.9, 95% CI 1.4-11.6; *P*=.01). Public respondents who were male (OR 2.11, 95% CI 1.26-3.61; *P*=.005), younger (OR 0.73 for a 10-year increase in age, 95% CI 0.60-0.89; *P*=.002), or spoke a language other than English at home (OR 1.99; 95% CI 1.09-3.59; *P*=.02) were more likely to have recorded a clinic visit. None of the large health systems we contacted reported a dedicated policy; however, 2 of the 49 health systems did report an existing policy that would cover the recording of clinic visits for patient use. The perceived benefits of recording included improved patient understanding and recall. Privacy and medicolegal concerns were raised.

**Conclusions:**

Policy guidance from health systems and further examination of the impact of recordings—positive or negative—on care delivery, clinician-related outcomes, and patients’ behavioral and health-related outcomes is urgently required.

## Introduction

Up to 80% of health care information discussed verbally is forgotten by patients after their clinic visit [1-4]. Poor recall and understanding of medical concepts have been identified as significant barriers to self-management, a central component of the chronic care model [5-7]. The last decade has seen significant efforts to increase patient access to medical information. Mandated through meaningful use, clinics across the United States now offer patients an after-visit summary (AVS) [8]. AVS is a summary of the clinic visit generated from the electronic medical record (EMR) available via the web-based patient portal, which includes information on diagnoses, medication, allergies, clinician visited, and summary of visit [9]. OpenNotes moves beyond this basic summary, offering patients access to the clinical notes in their EMR [10,11]. Access to such written summaries of office visits is associated with improved adherence, patient and caregiver satisfaction, patient self-care, medical information recall, and preparedness for clinic visits [11-16]. However, there have been concerns about the accuracy and complexity of written summaries [12-14] and their low use by patients [15]. This issue is compounded by low levels of health literacy; 35% of Americans have below basic or basic health literacy [16].

An adjunct to written summaries is the sharing of clinic visit audiorecordings with patients [17-20]. With broad and growing access to smartphones, recording devices are now ubiquitous, and reports of patients recording their clinic visits, with or without permission, are emerging [17,20]. Over 40 years of research finds that patient access to recordings results in greater patient understanding and recall of visit information, reduced decisional regret, and increased patient satisfaction [21-23]. Audiorecordings are also highly utilized in the research context; in a scoping review of 33 studies (18 trials), 71% of patients listened to their recordings and 68% shared them with a family member or caregiver [21]. In addition, according to a recent analysis, recording of clinic visits would be guided by “wire-tapping” laws, which premises that patients in 39 states and the District of Columbia, can legally make recordings without explicit consent of the clinician; the remaining 11 states require all party consent [19].

A handful of clinics in the United States have recognized the potential of recording and routinely offer patients audiorecordings (video recordings in one case) of their visits [18]. Furthermore, educational sessions are now available to clinicians in return for Continuing Medical Education credit for training in what to do if they find a “secret recording of office visits by patients” [24]. Despite this increased interest in recording, no data exist on the prevalence of recording in clinical practice in the United States or the attitudes of clinicians and the public toward recording. Additionally, it is unclear whether US hospital systems have created guidance or policies for clinicians and patients regarding the practice of recording. Such data are essential to assess the acceptability of recording and the potential of this strategy to become more widely implemented.

In this paper, we report on the prevalence of sharing audiorecorded clinic visits in the United States, the attitudes of clinicians and the public toward recording, and health system policies to guide recording practices.

## Methods

### Design

We administered 2 parallel cross-sectional surveys in July 2017 to US-based clinicians and the public. We also surveyed 49 of the largest health systems in the US by phone and email. All methods and materials were approved by Dartmouth College’s Committee for the Protection of Human Subjects (Study #30345). The usability and technical functionality of both surveys was tested by the research team and colleagues before fielding the surveys. We used the Checklist for Reporting Results of Internet E-Surveys to report our findings (see Multimedia Appendix 1).

### Participants

#### United States Clinicians

Clinicians were recruited and completed their surveys via SERMO (SERMO, Inc USA), the world’s leading online community of physicians who participate in online medical market research studies. SERMO has over 800,000 verified licensed physician members. To be eligible for inclusion, clinicians (Doctor of Medicine or Doctor of Osteopathic Medicine) had to be currently practicing in the United States. In order to recruit a diverse sample of specialties, we included clinicians from the following 8 specialties: emergency medicine, general or family medicine, internal medicine, general surgery, obstetrics-gynecology, orthopedic surgery, physical rehabilitation, and psychiatry. A bulk email was sent to a random sample of panel members from each specialty, informing them that they may be eligible to take part in a study. This voluntary, open survey consisted of 3 required multiple choice questions (clinician’s practice setting, years in practice, experiences of recording clinic visits) and 1 open response question assessing their views on patients having access to recordings (audio or video) of clinic visits and spanned over 1 screen (see Multimedia Appendix 2). Respondents could not review or change their answers or save responses if they wished to complete the survey later. Clinicians’ sociodemographic data (gender, age, practice location converted to Rural Urban Commuting Area codes) were available via SERMO. All data were collected over a one-day time period from July 6 to July 7, 2017.

#### United States Public

Participants were recruited online using Qualtrics Panels (Qualtrics LLC, Provo, Utah, USA). Quotas were applied based on US census data (gender, race, ethnicity, and language other than English at home) to approximate the US adult population [25-28]. A bulk email was sent to a random sample of panel members based on quotas, informing potential respondents that they may be eligible to take part in a study; however, no information on the content of the survey was provided until members “clicked” on the survey link. Respondents receive “points” from Qualtrics for taking part, which can be redeemed for an incentive, for example, air miles, gift cards, etc. To be eligible for inclusion, individuals had to be ≥18 years and reside in the United States. This voluntary, open survey consisted of 13 multiple choice questions (sociodemographics, experiences of recording clinic visits, and attitudes toward recording clinic visits) and 1 open response question assessing public views of recording clinic visits for patient's’ personal use (see Multimedia Appendix 3). All questions were required and adaptive questioning was used. The survey included 14 items and spanned over 3 screens. Respondents could not review or change their answers or save responses if they wished to complete the survey later. Data were collected over a one-week time period from July 13 to July 19, 2017.

In both clinician and public surveys, the recruitment invitation included general information about the study (approximate length, purpose, and investigators) and a link to the anonymous and confidential survey. Participants consented by their decision to continue onto the survey. To increase the quality of data in both surveys, responses from individuals who completed the survey in less than one-third of the median completion time were excluded. Only completed questionnaires were analyzed.

#### Health Care Systems

We identified 49 of the largest health systems in the United States using the employed physician counts in IQVIA’s OneKey reference data set and supplemented this with the information provided in the Agency for Healthcare Research and Quality (AHRQ) compendium of US Health Systems 2016 (see Multimedia Appendix 4) [29]. According to the AHRQ definition, “a health system includes at least one hospital and at least one group of physicians that provides comprehensive care (including primary and specialty care) who are connected with each other and with the hospital through common ownership or joint management.” Health care system administrators, specifically those who worked in risk management or other relevant areas, were contacted by email and asked whether they have a recording policy at their health system. If such a policy existed, they were asked to describe it. Nonresponsive systems were contacted by telephone 1 week later. A maximum of 3 phone calls and emails were made, after which the system was considered a nonresponder.

### Data Analysis

The prevalence of clinic visit recording and willingness to record was calculated for clinicians and the public. Multiple logistic regression analyses were conducted to identify factors associated with recording practices, including the history of recording, the history of covert recording (public respondents only), and willingness to record in the future. We planned to recruit a sample of 500 members of the public, which in a similarly sized probability sample would provide 95% CI of estimating the prevalence of recording in the population to within ±4%. We also aimed to sample at least 50 clinicians from each specialty, allowing for a minimum of 5 observations per parameter in the multiple logistic regression model [30]. Analyses were conducted using RStudio, V1.1.383 (RStudio, Boston, MA). We conducted a thematic analysis of all open-ended responses to identify salient themes reflecting the respondents’ attitudes toward patient recordings, as well as any concerns or related benefits. Comments were independently reviewed, and 20% were double coded by 2 members of our research team (MAD, KV). Finally, we categorized health systems as having an existing policy (and describing this policy), lacking an existing policy, or being unsure of their policies regarding clinic visit recordings.

## Results

### Clinician Survey

**Figure 1:**
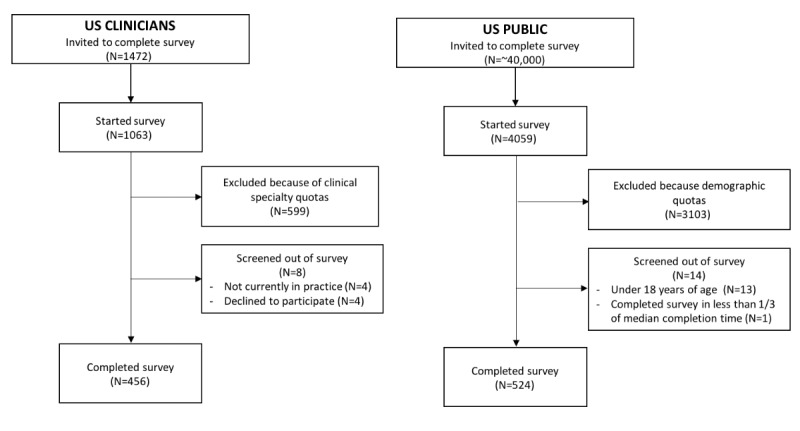
Overview of clinician and public survey participants.

A total of 1472 clinicians were invited to complete the survey, of which 409 did not respond (see Figure 1). Of the remaining 1063 clinician, 456 clinicians completed the survey, while 599 were excluded as the quotas for these clinicians’ specialties had been reached, and 8 clinicians were screened out (4 not currently in clinical practice and 4 declined). Respondents in the final sample (N=456) came from 44 states and Washington DC. Survey completion took an average of 2 minutes 30 seconds. Of the included respondents, 61% had been practicing for more than 10 years, and the majority, 84%, practiced at least half of their time in outpatient care (Table 1).

#### Prevalence of Recording Practices

Of 456 clinician respondents, 28.3% (129/456; 95% CI 24.2-32.7) reported that they had recorded a clinic visit for patients’ personal use (Table 2). Of the remaining 327 clinicians who had not recorded, 49.5% (162/327, 95% CI 44.0-55.1) were willing to do so, while 50.5% (165/327; 95% CI 45.0-56.0) were not. Multiple logistic regression analyses revealed that only clinical specialty was associated with recording a visit in the past (Table 3): clinicians in oncology and physical rehabilitation were more likely to have had a visit recorded (reference category, general or family medicine), odds ratio (OR) 5.1 (95% CI 1.9-14.9; *P*=.002) and OR 3.9 (95% CI 1.4-11.6; *P*=.01) respectively, and to be willing to be recorded by their patients in the future, OR 2.9 (95% CI 1.2-7.4; *P*=.02) and OR 2.9 (95% CI 1.2-7.6; *P*=.03), respectively (Table 3). Psychiatrist were also more willing to be recorded by their patients in the future, OR 2.7 (95% CI 1.1-6.8; *P*=.03).

**Table 1:** Clinician respondent characteristics (N=456).

Characteristics		n (%)
**Gender**		
	Female	99 (21.7)
	Male	291 (63.8)
	Chose not to answer	66 (14.5)
**Years in practice**		
	<5	63 (13.8)
	6-10	113 (24.8)
	11-15	84 (18.4)
	>15	198 (43.4)
**Clinical practice setting**		
	All inpatient care	19 (4.2)
	Mostly inpatient care	54 (11.8)
	Half inpatient care, half outpatient care	120 (26.3)
	Mostly outpatient care	151 (33.1)
	All outpatient care	112 (24.6)
**Location**		
	Urban	426 (93.4)
	Rural	29 (6.4)

**Table 2:** Clinician recording practices (N=456).

Specialty	Recording history, n (%)		
	Yes, I have had a visit recorded for a patient’s personal use	No, I have not had a visit recorded, however I would consider having a visit recorded in the future	No, I have not had a visit recorded and I would not consider having a visit recorded in the future
All (n=456)	129 (28.3)	162 (35.5)	165 (36.2)
Emergency medicine (n=51)	11 (21.6)	22 (43.1)	18 (35.3)
General or family practice (n=50)	8 (16.0)	15 (30.0)	27 (54.0)
General surgery (n=52)	18 (34.6)	16 (30.8)	18 (34.6)
Internal medicine (n=50)	11 (22.0)	17 (34.0)	22 (44.0)
Obstetrician-gynecologist (n=50)	14 (28.0)	18 (36.0)	18 (36.0)
Oncology (n=50)	23 (46.0)	13 (26.0)	14 (28.0)
Orthopedic surgery (n=51)	11 (21.6)	20 (39.2)	20 (39.2)
Physical rehabilitation (n=50)	21 (42.0)	17 (34.0)	12 (24.0)
Psychiatry (n=52)	12 (23.1)	24 (46.2)	16 (30.8)

**Table 3:** Characteristics of clinicians associated with having had a clinic visit recorded for a patient’s personal use in the past and willingness to have a clinic visit recorded for a patient’s personal use.

Factors		History of recording clinic visit for patient, OR^a^ (95% CI)	Willingness to record clinic visit in the future, OR (95% CI)
Gender (reference: female)		0.99 (0.58-1.75)	0.77 (0.44-1.30)
Years in practice (5 year increments)		1.03 (0.82-1.29)	0.83 (0.66-1.04)
**Setting (reference: ½ inpatient, ½ outpatient)**			
	Mostly inpatient	0.84 (0.38-1.78)	1.21 (0.57-2.64)
	Mostly outpatient	0.83 (0.46-1.49)	0.76 (0.43-1.34)
Location (reference: urban)		1.25 (0.48-2.99)	0.66 (0.29-1.50)
**Specialty (reference: general or family practice)**			
	Emergency medicine	1.72 (0.58-5.37)	2.10 (0.85-5.31)
	General surgery	2.85 (0.97-9.00)	1.75 (0.67-4.64)
	Internal medicine	1.12 (0.34-3.68)	1.65 (0.67-4.14)
	Obstetrician-gynecologist	2.25 (0.81-6.68)	1.88 (0.80-4.52)
	Oncology	5.11 (1.93-14.90)	2.90 (1.19-7.35)
	Orthopedic surgery	1.37 (0.41-4.66)	1.10 (0.41-2.95)
	Physical rehabilitation	3.91 (1.43-11.63)	2.90 (1.16-7.59)
	Psychiatry	1.69 (0.59-5.09)	2.75 (1.14-6.84)

^a^OR: odds ratio.

**Table 4:** General clinician and public attitudes toward patient access to audiorecordings of clinic visits.

Views on patient access to recordings of clinic visits	Physicians, n (%)	General public, n (%)
Total respondents who shared general attitude in open-ended response	377 (81.8)	459 (87.8)
Supportive of patient access to recordings	136 (36.1)	301 (65.6)
Supportive of case-by-case basis of recordings	34 (9.0)	11 (2.4)
Concerned about patient access to recordings	183 (48.5)	90 (19.6)
Uncertain, had never previously considered patient access to recordings	16 (4.2)	23 (5.0)
Neutral, no opinion toward patient access to recordings	8 (2.1)	34 (57.4)

#### Clinicians’ Views of Sharing Recordings of Clinic Visits

In the open-ended responses to their views on providing clinic recordings to patients (n=377), 170 clinicians were supportive of recording, 183 were concerned about recording, while the remaining clinicians were neutral or uncertain (Table 4). Proposed benefits included improved information recall and understanding, the ability for clinicians to use the audiorecordings for documentation purposes, and clinical education:

There are legitimate reasons to do it at times! Maybe for someone who is afraid they will not remember or for someone who could not be there.

I would welcome the idea if this replaces writing long notes on EMR.

It might be useful (in the right setting) as a tool for peer feedback on patient interaction.

Privacy concerns and risk of medicolegal use by patients emerged as the most common concerns among clinicians:

The recording of office visits would be used by lawyers to twist our words against us in court.

However, some clinicians considered it protective:

If I detect a potential litigious patient I would ask if the visit could be recorded.

Clinicians also expressed concerns regarding a negative impact on the patient-clinician interaction through potentially less “open” consultations and whether patients would use recordings:

...I'd be skeptical of how much patients would actually view the videos or benefit from the service.

### United States Public Survey

Approximately 40,000 individuals were invited to take part in the survey, of which 4059 responded (see Figure 1). Of those 4059 individuals, 524 completed the entire survey, while 3103 were excluded as quotas for these individuals were reached and 14 individuals were screened out (13 were under 18 years and 1 respondent completed the survey in less than one-third of the median completion time: 1 minute 48 seconds). Respondents in the final sample (n=524) belonged from 48 states. The sociodemographic characteristics of the respondents approximated that of the US population (Table 5).

#### Prevalence of Recording Practices

Of the public respondents, 15.6% (82/524; 95% CI 12.6-19.0) reported audio or video recording a clinic visit with permission, while 2.7% (14/524; 95% CI 1.5-4.4) did so secretly (Table 6). Additionally, 19.3% respondents (101/524; 95% CI 16.0-22.9) reported that they were aware of a family member or friend who reported recording a clinic visit, of which 60.4% (61/101; 95% CI 49.2-69.1) asked permission and 25.7% (26/101; 95% CI 17.6-35.4) did not. Finally, 58.6% (307/524; 95% CI 54.2-62.8) reported that they would consider recording a visit in the future with permission of the clinician and 7.4% (39/524; 95% CI 5.3-10.0) without the clinician’s permission, while 37.4% (196/524) were not interested in recording a clinic visit.

In a multiple logistic regression analysis, individuals who reported having recorded a clinic visit with the permission of their provider were more likely to be male, OR 2.11 (95% CI 1.26-3.61; *P*=.005); to be younger, OR 0.73 (95% CI 0.60-0.89; *P*=.002) per 10 years increase in age; and to speak a language other than English at home, OR 1.99 (95% CI 1.09-3.59; *P*=.02; Table 7). While 63% of general public respondents were interested in recording a clinic visit in the future, older adults (OR 0.88, 95% CI 0.78-0.99 per 10 years increase in age) and those with a lower level of education (OR 0.58, 95% CI 0.38-0.89) were less likely to be interested in recording a clinic visit for their personal use. This analysis did not reveal any demographic factors that were predictive of individuals having recorded a clinic visit covertly (all *P*>.15).

#### Public Views of Sharing Recordings of Clinic Visits

In the open-ended responses (n=459), 312 were supportive of recording and 90 were concerned about recordings, while the remaining public comments were considered neutral or uncertain (Table 6). Similar themes regarding concerns and potential benefits associated with recordings emerged from public respondents when compared to clinician respondents. The most common positive theme was the potential for recordings to improve patient recall and understanding of medical information:

I would like this option since I'm not very knowledgeable about medical terms and if I ask questions during the visit it might go over my head. If I can play it back, I would better absorb what I need to know or if I missed something, I can hear it again…

Additionally, a small proportion of respondents believed that recordings could be used for medicolegal purposes and to improve clinician recall of visit information:

It allows for patients and doctors to look back on the visit for information they might have missed.

Concerns were less common in the public sample, but included privacy concerns, *“These recordings could fall into the wrong hands* *”*; unclear benefit to patient of recordings, *“I'm not really sure what the point would be to have my clinic visits recorded...”*; and possible impact on the visit, *“recording my visits may inhibit my interaction with the health professional.”*

### Health Care System Recording Policies

When 49 of the largest health care organizations in the United States were asked in August 2017 about the existence of a policy regarding patient recording care systems, 47 responded to our request (Multimedia Appendix 4). Of the responses, 22 reported no formal policy, 13 were unsure if they had a policy, 4 stated that such policies would be left to the individual clinics, 6 said that the policy would be physician dependent, and 2 reported an existing policy that would cover patient requests for audiorecordings or videorecordings of the clinic visit (Table 8). Of the clinics that reported an existing policy that could be applied, the Henry Ford Health System, Michigan stated that patients’ audiorecordings or videorecordings and photographs must comply with privacy laws and their institutional policy.

**Table 5:** Public respondent characteristics (N=524).

Characteristics		n (%)
**Gender**		
	Female	255 (48.7)
	Male	262 (50.0)
	Other	7 (1.3)
**Age (years)**		
	18-40	304 (58.0)
	41-60	152 (29.0)
	>60	68 (13.0)
**Education**		
	High school degree or less	124 (23.7)
	Some college or college degree or equivalent	313 (59.7)
	Postgraduate degree (Masters, PhD, or professional)	73 (13.9)
	Other	14 (2.7)
**Hispanic origin**		
	Yes	66 (12.6)
	No	458 (87.4)
**Race**		
	American Indian or Alaska Native	4 (1.0)
	Asian	21 (4.0)
	Black or African American	75 (14.3)
	Native Hawaiian or other Pacific Islander	0 (0.0)
	White	412 (78.6)
	Other	12 (2.3)
**Language other than English spoken at home?**		
	Yes	104 (19.8)
	No	420 (80.2)

The Henry Ford Health System patient photographs and video recordings policy allows for recording, but consent must be attained first and the recording will be stored in the EMR, which patients can request access to. The Mayo Clinic, Minnesota stated that “with their consent, patients, families and staff may be photographed or video recorded by families and/or visitors at Mayo facilities for the purpose of education for continuing care of the patient following discharge,” but that no other forms of photography or video are allowed.

**Table 6:** Public recording practices (N=524).

Survey item		Respondent, n (%)
**Have you ever recorded (audio or video) a clinic visit with your doctor or health professional?**		
	Yes, and I asked for permission first	82 (15.6)
	Yes, and I did so secretly (without asking permission first)	14 (2.7)
	No, I have never recorded a clinic visit	431 (82.3)
**Would you consider recording a clinic visit with a doctor or another health professional?**		
	Yes, I would consider recording with the permission of the doctor	307 (58.6)
	Yes, I would consider secretly recording (without the permission of the doctor)	39 (7.4)
	No, I have no interest in recording a clinic visit	196 (37.4)
**Are recordings (audio or video) of patient clinic visits routinely offered in your clinic?**		
	Yes	51 (9.7)
	No	262 (50.0)
	Not sure	211 (40.3)
**Do you know a family member or friend who has recorded (audio or video) a visit with a doctor or health professional?**		
	Yes	101 (19.3)
	No	423 (80.7)
**Did the family member or friend ask permission before recording the clinic visit? (n=101)**		
	Yes	61 (60.4)
	No	26 (25.7)
	Not sure	14 (13.9)

**Table 7:** Characteristics of the public associated with a history of recording a clinic visit and with an interest in recording a clinic visit for their own personal use, with or without permission from their doctor or health professional.

Characteristics		History of recording with permission, OR^a^ (95% CI)	History of recording covertly, OR (95% CI)	Interest in recording, OR (95% CI)
**Age**				
	Increase of 1 year	0.97 (0.95-0.99)	1.01 (0.97-1.05)	0.99 (0.98-0.999)
	Increase of 10 years	0.73 (0.60-0.89)	1.10 (0.73-1.62)	0.88 (0.78-0.99)
Gender (reference: female)		2.11 (1.26-3.61)	1.55 (0.48-5.41)	1.22 (0.85-1.78)
**Education (reference: some college or college degree)**				
	High school or less	1.12 (0.61-2.01)	0.60 (0.09-2.36)	0.58 (0.38-0.89)
	Postgraduate degree	0.97 (0.41-2.08)	N/A^b^	0.75 (0.44-1.29)
Race (white non-Hispanic vs everybody else)		0.76 (0.43-1.36)	0.39 (0.11-1.45)	1.07 (0.69-1.66)
Language other than English spoken at home		1.99 (1.09-3.59)	1.49 (0.36-5.45)	1.54 (0.93-2.60)

^a^OR: odds ratio.

^b^N/A: not applicable.

**Table 8:** Responses from 49 large health care organizations in the United States.

Response	Organizations, n (%)
Has a policy	2 (4)
No policy	22 (45)
Policy is up to individual facilities	4 (8)
Policy is up to individual physicians	6 (12)
Unknown	13 (26.5)
No Response	2 (4)

## Discussion

### Principal Findings

In this study, the first to explore the prevalence of clinic recording in the United States, we found that one-third of surveyed clinicians have recorded a clinic visit for a patient's personal use and that half of those who have not recorded would be willing to do so in the future. Approximately one-fifth of the public reported recording a visit in the past and two-thirds would consider recording a visit in the future. Oncologists and physical rehabilitation clinicians were most likely to have recorded a visit. Members of the public who were younger, male, or spoke a language other than English at home were most likely to have recorded. Clinicians and patients commented on the benefits of recording, including improved recall and understanding. However, clinicians also reported privacy and medicolegal concerns. None of the 49 large health systems that we spoke to reported a dedicated policy or guidance for clinicians or patients on the practice of sharing clinic visit recordings; two reported that this would fall under an existing guideline.

### Limitations

This project is not without limitations. Since we used online panels to recruit clinicians and members of the general public, it is not possible to create a response rate. By ensuring that our respondent samples approximated census data with regard to age, gender, education, and language spoken at home, we reduced the potential impact of selection bias. Additionally, the representativeness of data gathered from internet panels has been shown to be comparable to that from probability-based general population samples [31]. We were not able to determine who instigated the recording for those who have recorded or whether this practice is routine in clinicians who reported sharing a recording in the past; however, 10% (51/524) of public respondents did report that this practice was routine at their clinic. Focusing on a sample of the public, rather than a sample of patients, may underrepresent the prevalence of recording occurring in health care as it includes a range of respondents, many of whom will have limited experience with health systems.

### Comparison with Prior Work

The current project supports previous findings that patients are beginning to “press record” during clinic visits [17-18,20]. Similar to previous studies, clinicians’ views on recordings were mixed. The benefits of increased understanding, recall, and the possibility of better self-management were tempered by medicolegal and privacy concerns. Despite these concerns, a significant proportion of clinicians have shared recordings or are willing to do so in the future.

Reports of covert recording in this project (14/524, 2.7%) are much lower than those reported in a previous study, where 15% of United Kingdom public respondents reported this practice [20]. This difference may be due to the high risk of selection bias in the UK survey, where a small convenience survey (n=128) was administered following a radio talk show discussing pros and cons of covertly recording clinic visits. Yet, in the present survey, 25.7% (26/101) of respondents who were aware of a family member or friend recording a visit reported that this was done covertly.

Only clinician specialty was associated with recording practice and intention to record, with almost half of oncologists and physical rehabilitation clinicians reporting that they had shared a recording in the past. Higher rates of recording in oncology may be due to the emotional nature of a cancer diagnosis and complex treatment plans. Additionally, most previous research on the use of recordings in health care has taken place in oncology settings [21]. While physical rehabilitation is less studied, the benefit of recordings in this population of patients has been documented in an ongoing case study (Barr; PJB, unpublished data, February 2017). Clinicians from general or family practice were the least likely (8/50, 16%) and least willing to record (15/50, 30%). Barriers to recording use among these clinicians may be due to the significant clinical informatics challenges reported, including the volume of clinical reminders and computerized patient record system alerts and time needed to input EMR notes [32]. Coupled with the diverse nature of patients and severity of conditions, it is not surprising that general and family clinicians are least likely to record. Yet, for these reasons, audiorecording could be beneficial in primary care, especially with advances in speech-to-text software that could assist with documentation at the point-of-care (see Implications).

Public respondents who spoke a language other than English at home were more likely to report recording a clinic visit. This may be a strategy to mitigate poor communication of health care information commonly reported by patients with low English proficiency [33-35]. Younger individuals also reported higher rates of recording, which may reflect their comfort with technology and greater likelihood of having a smartphone [36]. It is unclear why male patients are more likely to record than females, but this finding supports our previous survey in the UK [20]. The differences do not appear to be due to smartphone access or use [36]. It may be that because men are reported to delay health seeking compared with women, their clinic visits may be related to more complex problems where recording would be helpful [37]. Men may also be more likely to record in order to report back to women in their lives (eg, wife, sister, mother). Whereas women traditionally manage their family’s health care [38] and as such may feel less need to record and share their visit. Alternatively, they could simply be more willing to ask permission to record. Further investigation of individual differences in recording practice by minority groups and gender is required.

Further investigation of individual differences in recording practice by minority groups and gender is required.

### Implications

With no clear policies, it appears that clinicians and patients are leading the way on the implementation of recording in health care delivery. Through the lens of the “Diffusion of Innovation” model, the recent increase in the use of recordings is not unexpected as it meets the principles required for successful diffusion outlined by Berwick [39]: (1) the need for change is apparent; (2) the innovation is compatible with adopters’ values; (3) it is simple and flexible; (4) it is trialable; and (5) it is observable. The need to improve the transparency and communication of medical information in clinics is evident from recent policies, such as meaningful use [8] and advances in OpenNotes [10]. Furthermore, 40 years ago, recording of clinic visits was complicated, involving impractical technology; today, it is simple, so much so that clinics have many ways in which to implement recording practices. In a recent case study, clinics that routinely offered recordings used a range of approaches including patient phones, digital recorders, and clinicians’ computers to audiorecord and electronic tablets to videorecord visits [18]. It appears that we are at the early adopter stage of recording practice. The dissemination of innovations in health care has a tipping point of 15%-20% after which it is difficult to stop [40]. Recording and sharing of clinic visits may have reached this point.

With significant developments in fields of artificial intelligence and conversational analytics, health care will be transformed in the next decade; 35% of health care organizations plan to leverage artificial intelligence within 2 years and more than half intend to do so within 5 years [41-43]. Audiorecorded clinic data holds significant potential to tackle some of the major challenges we face today at lower costs, such as clinician documentation burden, patient recall of visit information, and improved patient-centered communication [44]. Highly accurate speech-to-text systems will enable real-time visit documentation [32]; patients and clinicians will once again be able to talk without the barrier of a computer. Our research group is developing a recording system that will use machine learning to tag key information from the clinic visit and link this to credible lay information for patients and their caregivers: Audio-Personal Health Library (PaHL) [45]. We hypothesize that Audio-PaHL will improve patient recall and understanding of visit information, resulting in improved self-management and a better health care experience via improved care coordination and higher satisfaction [18].

### Conclusions and Relevance

US clinicians and public are taking the lead on sharing clinic visit recordings, while policy makers lag behind. Policy guidance for clinics and further examination of the impact of recordings on clinical practice—both positive and potentially unforeseen negative—are urgently required.

## Appendix

Multimedia Appendix 1 Checklist for Reporting Results of Internet E-Surveys (CHERRIES).

Multimedia Appendix 2 National recording survey: clinicians.

Multimedia Appendix 3 National recording survey: general public.

Multimedia Appendix 4 Overview of health care systems.

## Abbreviations

AHRQ: Agency for Healthcare Research & Quality
AVS: after-visit summary
EMR: electronic medical record
OR: odds ratio
