# Supplementary material for: Audio-/Videorecording Clinic Visits for Patient’s Personal Use in the United States: Cross-Sectional Survey
Source: J Med Internet Res. 2018 Sep 12;20(9):e11308. doi: 10.2196/11308 (PMC6231772; doi:10.2196/11308)
Supplement: Multimedia Appendix 2 [file jmir_v20i9e11308_app2.pdf]

National Recording Survey: Clinicians

Dartmouth College

Patient and clinician experiences of recording clinic visits in the USA. A National Survey

Research Project Information Sheet

We are conducting a short survey about the recording (audio or video) and sharing of clinic visits with patients in the U.S. This project is led by Dr. Paul Barr from Dartmouth College, Hanover, New Hampshire, USA.

Your participation is voluntary. It involves completing a 2-minute survey. There are no right or wrong answers. You may choose to answer all or none of the questions, and your responses are anonymous and no identifiable data will be required.

If you have questions about this project you can contact:
Dr. Paul Barr, PhD
The Dartmouth Institute for Health Policy & Clinical Practice
Lebanon, NH 03756
paul.j.barr@dartmouth.edu

Screening question 1

S1. How many years have you been in practice? [select one]

❍ < 5 years
❍ 6-10
❍ 11-15
❍ > 15
❍ Not currently in practice (choosing this answer will end the survey)

Screening question 2

S2. Which of the following best describes your current clinical practice setting? [select one]

❍ All inpatient care
❍ Mostly inpatient care
❍ Approximately half inpatient care and and half outpatient care
❍ Mostly outpatient care
❍ All outpatient care
❍ I don’t currently practice in a clinical setting (choosing this answer will end the survey)

Q1. ***Have you ever had a clinic visit recorded (audio/video) for a patient's personal use?***
Select all that apply

- ❍ Yes, I have had a visit recorded for a patient's personal use
- ❍ No, I have not had a visit recorded, however I would consider having a visit recorded in the future
- ❍ No, I have not had a visit recorded and I would not consider having a visit recorded in the future

Q2. ***Briefly, what are your views about patients having access to recordings of clinical visits? Is this something routinely offered in your clinic?***
This information is important to us, please consider leaving your thoughts.
