# Supplementary material for: Audio-/Videorecording Clinic Visits for Patient’s Personal Use in the United States: Cross-Sectional Survey
Source: J Med Internet Res. 2018 Sep 12;20(9):e11308. doi: 10.2196/11308 (PMC6231772; doi:10.2196/11308)
Supplement: Multimedia Appendix 3 [file jmir_v20i9e11308_app3.pdf]

National Recording Survey: General Public

Dartmouth College
Patient and clinician experiences of recording clinic visits in the USA. A National Survey
Research Project Information Sheet

We are conducting a short survey about patients who audio or video record office visits with a doctor or other health professional. This project is led by Dr. Paul Barr from Dartmouth College, Hanover, New Hampshire, USA.

Your participation is voluntary and you must be 18 years of age or older to take part. It involves completing a 2-minute survey. There are no right or wrong answers. Your responses are anonymous and no identifiable data will be collected.

If you have questions about this project, you can contact:
Dr. Paul Barr, PhD
The Dartmouth Institute for Health Policy & Clinical Practice
Lebanon, NH 03756
paul.j.barr@dartmouth.edu.

If you wish to participate in this project, please click the ">>" button below
If you do not wish to participate in this project, or if you are under 18 years of age, please close your Internet browser.

Q1 What is your age?

____

Q2 **What state do you live in?**

▼ Alabama (1) ... United States Virgin Islands (56)

Q3 **What is your gender?**

O Male

O Female

O Other

Q4 **What is the highest level of schooling you have completed, or degree you have received?**

O No schooling completed, or less than 1 year

O Nursery, kindergarten, and elementary (grades 1-8)

O High school (grades 9-12, no degree)

O High school graduate (or equivalent)

O Some college (1-4 years, no degree)

O Associate’s degree (including occupational or academic degrees)

O Bachelor’s degree (BA, BS, AB, etc)

O Master’s degree (MA, MS, MENG, MSW, etc)

O Professional school degree (MD, DDC, JD, etc)

O Doctorate degree (PhD, EdD, etc)

O Other. Please specify: ____

Q5 **Are you of Hispanic, Latino, or Spanish origin?**

O I am of Hispanic, Latino, or Spanish origin

O I am NOT of Hispanic, Latino, or Spanish origin

Q6 **What is your race?**

- ○ American Indian or Alaska Native
- ○ Asian
- ○ Black or African American
- ○ Native Hawaiian or other Pacific Islander
- ○ White/Caucasian
- ○ Other. Please specify: ____

Q7 **Do you speak a language other than English at home?**

- ○ Yes
- ○ No

Q8 **Have you ever recorded (audio or video) a clinic visit with your doctor or health professional? (Select all that apply)**

- ☐ Yes, and I asked for permission first.
- ☐ Yes, and I did so secretly (without asking permission first)
- ☐ ⊗ No, I have never recorded a clinic visit.

Q9 **Do you know a family member or friend who has recorded (audio or video) a visit with a doctor or health professional?**

O Yes

O No

Display This Question:

If Do you know a family member or friend who has recorded (audio or video) a visit with a doctor or... = Yes

Q10 **Did the family member or friend ask permission before recording the clinic visit?**

O Yes

O No

O Not sure

Q11 **Would you consider recording a clinic visit with a doctor or another health professional? (Select all that apply)**

☐ Yes, I would consider recording with the permission of the doctor.

☐ Yes, I would consider secretly recording without the permission of the doctor.

☐ ⊗ No, I have no interest in recording a clinic visit.

Q12 **Are recordings (audio or video) of patient clinic visits routinely offered in your clinic?**

O Yes

O No

O Not sure

Display This Question:

If Are recordings (audio or video) of patient clinic visits routinely offered in your clinic? = No

Q13 **Would you like your clinic to allow you to record clinic visits with a doctor or another health professional?**

O Yes

O No

Q14 **Briefly, what are your views on having access to audio recordings of your clinic visit?** *This information is important to us, please consider leaving your thoughts.*

____

____

____

____

____
