# Supplementary material for: Audio-/Videorecording Clinic Visits for Patient’s Personal Use in the United States: Cross-Sectional Survey
Source: J Med Internet Res. 2018 Sep 12;20(9):e11308. doi: 10.2196/11308 (PMC6231772; doi:10.2196/11308)
Supplement: Multimedia Appendix 4 [file jmir_v20i9e11308_app4.pdf]

Supplemental Table 1. Forty-nine of the largest healthcare organizations in the USA, listed by number of clinicians given in the AHRQ Compendium of US Health Systems

**Name of system**	**State**	**Teaching Hospital(s)**	**Number of clinicians**	**% Primary Care Clinicians**
Kaiser Permanente	CA	Yes	20300	44%
Sutter Health	CA	Yes	10610	16%
Ascension Health	MO	Yes	10502	37%
Trinity Health	MI	Yes	8790	38%
Providence Health and Services	WA	Yes	8640	35%
University of California Office of the President Health Sciences and Services	CA	Yes	7402	25%
Partners HealthCare System, Inc	MA	Yes	7368	20%
Tenet Healthcare Corporation	TX	Yes	6297	25%
Mayo Clinic	MN	Yes	6127	25%
Catholic Health Initiatives	CO	Yes	5422	36%
MedStar Health	MD	Yes	4786	18%
Texas Health Resources	TX	No	4779	24%
Community Health Systems, Inc	TN	Yes	4283	37%
The Cleveland Clinic Health System	OH	Yes	4256	26%
Northwell Health	NY	Yes	3721	24%
UPMC	PA	Yes	3610	27%
Mount Sinai Health System	NY	Yes	3587	24%
University of Pennsylvania Health System	PA	Yes	3426	22%
New York Presbyterian Healthcare System	NY	Yes	3394	25%
Carolinas HealthCare System	NC	Yes	3339	40%
UW Medicine	WA	Yes	3164	23%
New York City Health and Hospitals Corporation	NY	Yes	3105	37%
Banner Health	AZ	No	3003	35%
University of Michigan Health System	MI	Yes	2795	22%
Baylor Scott and White Health	TX	Yes	2743	37%
SSM Health	MO	Yes	2608	34%
Henry Ford Health System	MI	Yes	2591	26%
Johns Hopkins Health System	MD	Yes	2549	30%
Mercy Health	MO	Yes	2540	33%
Advocate Health Care	IL	Yes	2496	40%
Indiana University Health	IN	Yes	2484	31%
Dignity	CA	Yes	2403	36%
Montefiore Medical Center	NY	Yes	2386	34%

Supplemental Table 1. Cont’d

**Name of system**	**State**	**Teaching Hospital(s)**	**Number of clinicians**	**% Primary Care Clinicians**

University of Maryland Medical System	MD	Yes	2027	26%
BJC HealthCare	MO	Yes	2021	29%
Universal Health Services, Inc	PA	Yes	1989	23%
Adventist Health System	FL	No	1891	39%
Beaumont Health Systems	MI	Yes	1818	33%
PeaceHealth	WA	No	1751	37%
LifePoint Health	TN	No	1699	39%
RWJBarnabas Health	NJ	Yes	1590	29%
Novant Health, Inc	NC	No	1461	46%
CHRISTUS Health	TX	No	1444	33%
Mercy Health	OH	Yes	1423	40%
Prime Healthcare Services, Inc	CA	Yes	1336	24%
Presence Health	IL	Yes	1153	40%
Hospital Corporation of America, Inc*	--	--	--	--
Veterans Health Administration*	--	--	--	--
TRICARE Regional Office North*	--	--	--	--

*Indicates large systems that are included in HCOS but not included in the AHRQ Compendium of US Health Systems because they do not fit the AHRQ working definition of a health system, e.g. they do not include a medical group, or do not have at least one non-Federal general acute care hospital
